# Hypertensive Response to Exercise as an Early Marker of Disease Development

**DOI:** 10.3390/biomedicines13010030

**Published:** 2024-12-26

**Authors:** Wojciech Kosowski, Krzysztof Aleksandrowicz

**Affiliations:** 1 Institute of Heart Diseases, Wroclaw Medical University, Borowska 213, 50-556 Wroclaw, Poland; 2 Institute of Heart Diseases, University Hospital, Borowska 213, 50-556 Wroclaw, Poland; krzysztof.aleksandrowicz@umw.edu.pl; 3 University Center for Physiotherapy and Rehabilitation, Faculty of Physiotherapy, Wroclaw Medical University, Chalubińskiego 3, 50-368 Wroclaw, Poland

**Keywords:** hypertension, elevated blood pressure, hypertensive response to exercise, exaggerated blood pressure response to exercise

## Abstract

Arterial hypertension is one of the world’s leading risk factors for death and disability. With the number of people living with this disease doubling between 1990 and 2019 from 650 million to 1.3 billion, it is a global burden that increases mortality from cardiovascular and kidney diseases. It is extremely important to use all possible diagnostic methods, indicating the possibility of early detection that subsequently leads to effective prevention of disease development. The phenomenon called hypertensive response to exercise (HRE) is one such method. The HRE is defined as a pathological, excessive increase in blood pressure as a result of exposure to the stressor, which is physical exercise. There is no consensus about precise cutoffs in the definition of this condition, which is most commonly diagnosed based on systolic blood pressure (SBP) ≥ 210 mm Hg in men and ≥190 mm Hg in women at peak exercise intensity. The fact that exercise hypotension is a pathologic sign is universally accepted. Accumulating data deliver the information that HRE is also connected to higher overall cardiovascular risk. It was demonstrated that HRE is associated with functional and structural impairment of the left ventricle and the future development of hypertension. HRE should act as a warning signal of increased cardiovascular risk, leading to the need for profound clinical care.

## 1. Arterial Hypertension—Epidemiology, Pathophysiology, and Clinical Consequences

Arterial hypertension (HT) is becoming an ever-increasing challenge for healthcare systems around the world, which is clearly supported by epidemiological data. Hypertension, with the number of people living with this disease doubling between 1990 and 2019 from 650 million to 1.3 billion, is a global burden that increases mortality from cardiovascular and kidney disease [1]. It is estimated that among adults, the prevalence of hypertension in the United States will increase from 51.2% in 2020 to 61.0% in 2050 [2]. A global report on hypertension prepared by the World Health Organization emphasized that high blood pressure is one of the world’s leading risk factors for death and disability [3]. In the Global Burden of Diseases 2019 study, hypertension was ranked as the top risk factor contributing to cardiovascular diseases between 1990 and 2019 [4].

Several interactions between environmental, behavioral, socio-economic and psychosocial factors, genes, hormonal networks, cardiovascular, renal, and central nervous systems, and vascular and immune mechanisms are involved in the pathophysiology of hypertension. A number are listed in the 2024 ESC Guidelines for the management of elevated blood pressure and hypertension:

- Geopolitical status, noise pollution, air pollution, climate;
- Physical activity, sedentary behavior, sleep quality and quantity, dietary patterns, sodium and potassium intake, obesity, alcohol consumption, drugs or substances that increase blood pressure;
- Stress, low socio-economic status, social deprivation, healthcare access, gender identity, roles and norms, gender-based violence, discrimination;
- Biological sex, BP-associated single-nucleotide polymorphisms, monogenic forms of hypertension, epigenetic and fetal programming;
- Renin–angiotensin–aldosterone system;
- Endothelial dysfunction, small artery remodeling, large artery stiffness;
- Salt sensitivity, pressure-natriuresis, renal ischemia;
- Autonomic nervous system, baroreceptor reflex [5].

When arterial hypertension is uncontrolled, it can lead to several hypertension-mediated organ damages (HMOD) affecting the following:

1. Heart: left ventricular remodeling and hypertrophy (both of which influence left ventricular diastolic and systolic function); increased left ventricular pressure, which promotes left atrial dilatation and dysfunction (ultimately leading to atrial fibrillation development); hypertension also contributes to coronary artery disease (including myocardial infarction);
2. Large and medium arteries: atherosclerosis, vascular calcification, arterial stiffness;
3. Microcirculation: endothelial dysfunction, increasing vasoreactivity, vascular remodeling, fibrosis and inflammation, increasing vascular resistance;
4. Kidney: glomerular arteriolar hypertension, glomerulosclerosis, albuminuria, and proteinuria, decrease in glomerular filtration rate;
5. Eye: microvascular remodeling, hypertensive retinopathy;
6. Brain: it is an early target of HMOD, which may manifest in such diseases as stroke, transient ischaemic attack, and also cognitive decline. Hypertension-induced small vessel disease can lead to both morphological (leucoaraiosis, white matter lesions, brain atrophy) and clinical complications (lacunar infarction, cerebral hemorrhage) [5].

If left untreated, HMOD usually progresses gradually from asymptomatic to symptomatic, ultimately ending with overt cardiovascular disease (CVD) events [5]. The above-mentioned complications are related also to the length of time during which blood pressure was not sufficiently controlled therapeutically.

Therefore, it is extremely important to use all possible diagnostic methods, indicating the possibility of early detection that subsequently leads to effective prevention of the disease. The phenomenon called hypertensive response to exercise (HRE) is one such method.

## 2. Physiology of Cardiovascular Response to Exercise

In health, exercise requires several adaptive mechanisms for effective, long-lasting muscle work. Those mechanisms allow fast switching between a resting and an excited state, which allows us to start any exercise immediately as soon as we need to. This primitive feature was crucial for the survival of our species and evolution, as it allowed us to adapt and react to several external stressors. The cardiovascular system adapts to exercise at various levels.

First and foremost, the cardiac output must be quickly increased without a decrease in systemic vascular resistance (as a natural consequence), which is achieved through changes in various aspects of the cardiovascular system. Moreover, the total circulatory blood also needs to be quickly augmented (recruited from a splanchnic bed), muscle perfusion must be maintained, and several additional cellular and molecular adaptations are inevitable.

In healthy people, systolic blood pressure (SBP) physiologically increases gradually with increasing exercise intensity, while diastolic blood pressure (DBP) may change only slightly or even fall during exercise [6].

Cardiac output (CO) increases to fulfill the working muscles’ greater metabolic requirements. CO augmentation results from increased stroke volume (SV) and heart rate (HR). From a mathematical point of view, CO = HR × SV. In a healthy individual, CO increases are accompanied by modest increases in both mean BP and pulse pressure [7].

At rest, the heart pumps approximately 5–6 L of blood per minute in an adult (cardiac output). During physical exercise, the capacity of blood pumped by the left heart into the arterial reservoir may increase up to seven times, reaching ~35 L/min. At the same time, cardiac stroke volume can increase at most three-fold, indicating that the enormous increase in cardiac output during exercise results from coordinated cooperation between increased heart rate, cardiac output, and vascular tone control. The blood vessels through which blood flows are not just “passive pipes” but play an important role in blood circulation. During intense physical exercise, changes occur in the contraction rate, stroke volume, and peripheral resistance. Cardiac output increases and total peripheral resistance decreases due to the dilation of arterioles in the muscles. These two changes have opposite effects on mean arterial pressure, but because cardiac output increases more than peripheral resistance decreases over time, mean arterial pressure ultimately increases [8].

The pressures in the arterial system show great changes during exercise. There is an increase in systolic and mean pressure in proportion to the intensity of the effort and oxygen consumption. Since the diastolic pressure remains essentially unchanged, the pulse pressure also increases in proportion to the intensity of the effort. The pressures in the venous system, both peripheral and central, show no great changes, but the blood flow in the veins increases and the return of venous blood to the heart increases as a result of the increased tonus of the venous vessels under the influence of the increase in sympathetic vasoconstrictor activity. This increase in activity may cause a transient increase in venous pressure at the beginning of the effort. The pressure in the pulmonary circulation shows a slight increase [8].

**Figure 1:**
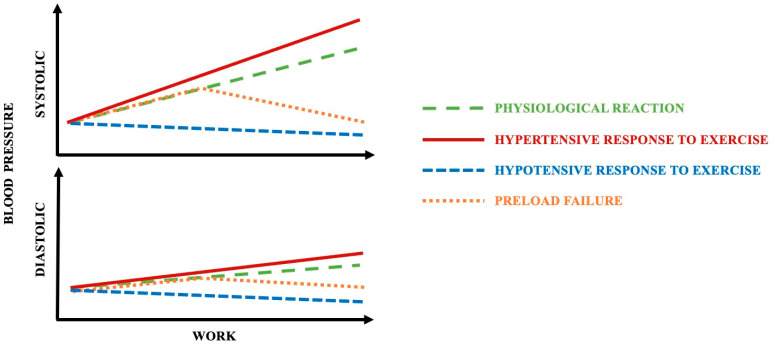
A schematic representation of the possibilities of changes in blood pressure during physical exercise.

The possibilities of changes in blood pressure during physical exercise are summarized in Figure 1. One of the possibilities contains the conception of preload reserve and, connected with it, preload failure. Preload reserve plays a relevant role in a normal physiologic response, and it is required to fulfill the changing metabolic demands. In a healthy individual, the recruitment of preload leads to an increase in effective circulating blood volume and as a result, an increase in cardiac output is observed [9]. It is believed that the abnormalities of cardiac preload reserve are a common cause of exercise limitations [10]. In a situation of increased cardiac demand during exercise, preload reserve failure limits CO and as a result leads to exercise limitations [11].

All of the above-described relations may be crucial for better understanding the key message of our paper. Those individuals whose cardiovascular control is not able to decrease vascular resistance enough during exercise (clinically identified as those with HRE) may represent the population of patients with early cardiovascular system control dysfunction. Thus, pathologically high blood pressure as a response to exercise may be an early marker of control dysfunction.

**Figure 2:**
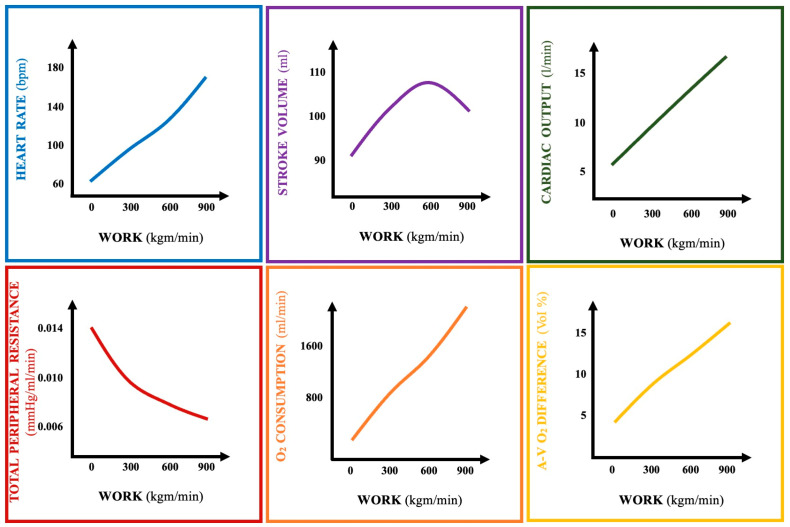
Changes in heart rate, stroke volume, cardiac output, total peripheral resistance, oxygen consumption, and arteriovenous O_2_ difference in relation to the amount of work performed (A-V O_2_ DIFFERENCE = arteriovenous oxygen difference).

The changes in heart rate, stroke volume, cardiac output, total peripheral resistance, oxygen consumption, and arteriovenous O_2_ difference in relation to the amount of work performed are summarized in Figure 2.

## 3. Hypertensive Response to Exercise (HRE)—Definition, Nomenclature, and Prevalence

Despite the proper blood pressure (BP) values while measuring at rest, some people may experience excessive increases in BP during exercise, a condition known as a hypertensive response to exercise (HRE).

HRE is defined as a pathological, excessive increase in blood pressure as a result of exposure to the stressor, which is physical exercise.

There is no consensus about the cutoffs to diagnose HRE. It is most commonly diagnosed based on SBP ≥ 210 mm Hg in men and ≥190 mm Hg in women at peak exercise intensity [12]. Taking into account diastolic blood pressure (DBP), a value ≥ 110 mm Hg for both sexes is considered the threshold for the diagnosis of HRE [12]. In some publications, HRE was also defined in another way—as a difference of 60 mm Hg between baseline and peak SBP for men and 50 mm Hg for women [13].

Currently, there is an ongoing trial adopting a new definition of the hypertensive response to physical exercise: SBP ≥ 150 mm Hg in the early phase of the exercise test, i.e., at stage 1 or 2 of the Bruce protocol. This has been called an exaggerated blood pressure response to exercise at moderate exercise intensities (ExBPR-MI). The idea behind adopting the new definition is to be able to reliably assess a larger number of patients, including those for whom it would be impossible to achieve the next stages of the Bruce protocol. Some studies adopting a similar concept have already been published.

There are many interchangeably used terms in the literature to describe this phenomenon. Hypertensive response to exercise (HRE) seems to be the most common one, but exaggerated blood pressure response (EBPR) or exaggerated exercise blood pressure (EEBP) are also frequently used.

A summary of the differences in HRE definitions is provided in Table 1. Table 2 presents the advantages and disadvantages of both definitions of HRE (peak exercise intensity vs. moderate exercise intensity).

The prevalence of this phenomenon has previously been suggested to be 5% to 40% [13]. HRE is even more common when considering patients with type 2 diabetes mellitus, reaching more than 50% [12].

## 4. Hypertensive Response to Exercise (HRE)—The Predictive Factors of HRE, the Predictive Significance for New-Onset Hypertension, and the Independent Association with CV Outcomes

Based on previous reports, it is known that HRE is a particularly common phenomenon in patients with elevated resting BP, masked hypertension, and type 2 diabetes mellitus [7].

As has been demonstrated, HRE is a phenomenon that predicts future incidence of hypertension. Still, with its association with cardiovascular events and mortality, the clinical relevance of HRE is even more important [14]. The first meta-analysis demonstrating the importance of HRE in predicting incident hypertension independently of resting BP and other cardiovascular risk factors is worth mentioning. Schultz et al. searched seven online databases for studies measuring dynamic exercise BP that reported incident hypertension among those normotensives at baseline. The analysis of a total of 23,207 participants provided evidence supporting the clinical value of HRE in detecting BP-related cardiovascular risk that would otherwise go undetected by conventional resting BP measurements [16]. What seems to be of the most relevance, patients with HRE compared with those with diagnosed hypertension, presented similar end-organ damage (including diastolic dysfunction, left ventricular hypertrophy. or albuminuria) [7].

Research results on the importance of HRE are presented in the subsection Relevance of HRE—Studies Review. Considering all the results, HRE should act as a warning signal of increased cardiovascular risk, leading to the need for profound clinical care.

## 5. Potential Mechanisms of Hypertensive Response to Exercise

Although the physiological causes of increased blood pressure due to physical exercise are well known, the pathophysiology of HRE still needs to be fully understood.

Among the mechanisms of HRE are mentioned augmented reflex pressor responses through enhanced activation of metaboreceptors increase sympathetic vasoconstriction in exercising muscle, which leads to a nonproportional increase in systemic vascular resistance, decreased nitric oxide and prostaglandin bioavailability [7].

As a result of adrenergic stimulation, part of the fluid volume is recruited from the visceral vascular system (which is necessary to ensure adequate CO and perfusion). However, fluid redistribution is a phenomenon that is also described in heart failure, especially heart failure with preserved ejection fraction (HFpEF), and it also contributes to the development of congestion [17].

The study from the Johns Hopkins School of Medicine suggests that one of the mechanisms contributing to exercise hypertension is impaired endothelial vasodilator function. It may also act as a link between exaggerated exercise BP and worsening hypertension [18]. Authors Kim and Ha also stated that HRE can be explained by the impairment of exercise-induced endothelial vasodilation. They stated that while endothelial dysfunction primarily contributes to HRE in younger individuals, arterial stiffness should be considered as a mechanism for HRE in older individuals. In support of the latter, a study demonstrated a positive association of HRE with large artery stiffness [19].

The results of another study suggest that inflammation may also be associated with HRE [20].

Other studies demonstrated the links between HRE and the sympathetic nervous system, the renin–angiotensin–aldosterone system, abnormal glucose metabolism, insulin resistance, or endothelium-derived hyperpolarizing factors [19].

Taking these studies together, it seems very likely that several mechanisms conspire together to lead to the development of the phenomena.

## 6. Exercise Blood Pressure Measurement Technique and Obstacles

It may seem challenging and difficult to measure BP during exercise and avoid mistakes connected to technical obstacles related to the movement of the patients and the noise of a running treadmill or cycloergometer. Aware of these difficulties, Sharman and LaGerche described an exercise BP measurement technique [7]. The general rules are as follows. It is essential to use manual cuff auscultation or a validated automatic BP device (in this case, it is important to test reliability by comparison with manual cuff auscultation before routine use) with a variety of available cuff sizes. The position of the BP monitor should be within 1 m of the operator, and the gauge should be viewed straight on. It is also important to avoid excess stethoscope pressure and measure BP each time with the cuff supported at the heart level, shoulder/arm relaxed, and no talking during pre-exercise measures. The person performing the test should explain to the patients not to grip the treadmill rails tightly. The authors also paid attention to following calibration and maintenance procedures for the BP device according to the manufacturer’s directions and comparing readings of automated devices with manual cuff auscultation every month.

### Indications for Terminating Exercise Testing

Indications for terminating exercise testing can be divided into absolute and relative. Among absolute indications are: ST-segment elevation (>1.0 mm) in leads without preexisting Q waves because of prior MI (other than aVR, aVL, and V1), drop in systolic blood pressure >10 mmHg, despite an increase in workload, when accompanied by any other evidence of ischemia, moderate-to-severe angina, central nervous system symptoms (e.g., ataxia, dizziness, near syncope), signs of poor perfusion (cyanosis or pallor), sustained ventricular tachycardia (VT) or other arrhythmias, including second- or third-degree atrioventricular (AV) block, that interferes with normal maintenance of cardiac output during exercise, technical difficulties in monitoring the ECG or systolic blood pressure, and the subject’s request to stop.

Relative indications for terminating exercise testing are as follows: marked ST displacement (horizontal or downsloping of >2 mm, measured 60 to 80 ms after the J point [the end of the QRS complex]) in a patient with suspected ischemia, drop in systolic blood pressure > 10 mmHg (persistently below baseline) despite an increase in workload, in the absence of other evidence of ischemia, increasing chest pain, fatigue, shortness of breath, wheezing, leg cramps, or claudication, arrhythmias other than sustained VT, including multifocal ectopy, ventricular triplets, supraventricular tachycardia, and bradyarrhythmias that have the potential to become more complex or to interfere with hemodynamic stability, exaggerated hypertensive response (systolic blood pressure > 250 mmHg or diastolic blood pressure > 115 mmHg), and development of bundle-branch block that cannot immediately be distinguished from VT [21].

## 7. Guidelines

We analyzed the guidelines on hypertension for information on HRE, taking into account European, American, Chinese, Japanese, Canadian, and British guidelines.

Current 2024 European Society of Cardiology (ESC) Guidelines for the management of elevated blood pressure and hypertension do not devote much attention to the hypertensive response to exercise [5]. However, it was underlined that an exaggerated BP response to exercise may have diagnostic value in predicting incident hypertension and CVD. Two publications were mentioned. In one of them, a meta-analysis, an exaggerated BP response to exercise was associated with an increased risk for masked hypertension [22]. The second publication stated that the risk of coronary heart disease also increases with higher systolic BP during exercise, independent of systolic BP at rest [23].

There is even less information regarding HRE in the 2017 American College of Cardiology (ACC)/American Heart Association (AHA)/AAPA/ABC/ACPM/AGS/APhA/ASH/ASPC/NMA/PCNA Guideline for the Prevention, Detection, Evaluation, and Management of High Blood Pressure in Adults. This Guideline just marks that patients with HFpEF have an exaggerated hypertensive response to exercise [24]. Although these guidelines are already 7 years old, the little attention paid to the problem of HRE proves that there is still ample scope for research proving the clinical importance of this phenomenon.

There is no information regarding HRE in the 2014 Evidence-Based Guideline for the Management of High Blood Pressure in Adults—Report From the Panel Members Appointed to the Eighth Joint National Committee (JNC 8) [25].

Similarly, no HR-related data can be found in the 2018 Chinese Guidelines for Prevention and Treatment of Hypertension—A report of the Revision Committee of Chinese Guidelines for Prevention and Treatment of Hypertension [26].

In the Japanese Society of Hypertension Guidelines for the Management of Hypertension (JSH 2019), which are over 200 pages long, there is only one mention saying that in normotensive women, intervention for exercise and diet resulted in alleviation of marked blood pressure elevation immediately after exercise, accompanied by improvement in pulse wave velocity (PWV) and alleviation of plasma nitric oxide (NO) level elevation [27].

No information on HRE can be found in Hypertension Canada’s 2020 Comprehensive Guidelines for the Prevention, Diagnosis, Risk Assessment, and Treatment of Hypertension in Adults and Children [28].

The National Institute for Health and Care Excellence Guidelines—Hypertension in adults: diagnosis and management do not provide any information on HRE either [29].

A summary of HRE data across guidelines is presented in Table 3.

## 8. Relevance of HRE—Studies Review

High exercise systolic blood pressure was reported to be associated with left ventricular hypertrophy (LVH) as early as the 1980s [30,31]. The data from one of the studies suggested that SBP achieved at a low level of exercise (five metabolic equivalents; METs) may be the most important determinant of LVH in patients with HT [30]. Another study concluded that patients with an exercise SBP of ≥190 mmHg usually have an increased LV mass [31]. Although these data seem to provide important clinical information, they require further in-depth research.

Schultz, Picone, Nikolic, Williams, and Sharman hypothesized in their study that HRE may indicate hypertension undetected by standard clinical resting BP measurements but noted that this has never been confirmed by an association with hypertension defined by ambulatory BP monitoring, which was the aim of their study. One hundred consecutive patients without the diagnosis of coronary artery disease (aged 56 ± 9 years, 72% male) underwent clinically indicated exercise testing. Exercise BP was recorded at each step of the Bruce protocol. The presence of hypertension was defined as 24 h SBP ≥ 130 mm Hg or daytime SBP ≥ 135 mmHg. Stage 1 and stage 2 exercise systolic BP were significantly associated with the presence of hypertension, with the strongest association observed between stage 1 exercise SBP and 24 h SBP > 130 mmHg. A total of 79% of participants who achieved SBP ≥ 150 mmHg at the first stage of the test were classified as having hypertension, with SBP > 150 mmHg predicting hypertension regardless of age, sex, and clinic hypertension status. It was summarized that irrespective of resting BP, SBP ≥ 150 mmHg during the early stages of the Bruce exercise stress test was associated with the presence of hypertension. The researchers emphasized that HRE should be a warning signal to health professionals about the presence of hypertension and the need to provide follow-up care to reduce cardiovascular risk [14].

Kokkinos, Pittaras, Narayan, Faselis, Singh, and Manolis assessed echocardiographic and exercise parameters in 790 prehypertensive individuals to determine associations between exercise blood pressure and left ventricular structure. The exercise SBP at five metabolic equivalents (METs) and the change in blood pressure from rest to five METs were the strongest predictors of left ventricular hypertrophy. Both identified the SBP of 150 mmHg at exercise levels of five METs as the threshold for left ventricular hypertrophy [32].

The use of the above threshold as the definition of HRE made it possible to assess the BP response in a larger group of patients, including those who would not be able to achieve the more advanced stages of the Bruce protocol and, consequently, a higher level of exercise due to cardiac (chronotropic insufficiency) and non-cardiac (deconditioning, musculoskeletal disorders) limitations. Another advantage of the above definition of HRE is that moderate exercise intensity better corresponds to routine daily activities than peak exercise intensity, which has been considered in other studies. Furthermore, the relatively slower speed of the treadmill during the early stages of the examination is associated with less movement of the patient’s arm and less interference with auscultation of blood pressure, allowing for a more accurate assessment.

Schultz, Otahal, Cleland, Blizzard, Marwick, and Sharman, in their meta-analysis, identified for review 12 longitudinal studies with a total of 46,314 individuals without significant coronary artery disease, with total cardiovascular event and mortality rates recorded over a mean follow-up of 15.2 ± 4.0 years. After adjustment for age, office BP, and CV risk factors, the HRE at moderate exercise intensity was associated with a 36% higher rate of CV events and mortality than in people without HRE [6].

Weiss, Blumenthal, Sharrett, Redberg, and Mora assessed a total of 6578 asymptomatic Lipid Research Clinics Prevalence Study participants performing submaximal Bruce treadmill tests who were followed for 20 years. Bruce stage 2 BP 180/90 mmHg identified non-hypertensive individuals at higher risk of CVD death [33]. This emphasizes the vital information one can learn from the test and highlights the clinical importance of preclinical hypertension and hypertensive response to exercise.

The aim of another investigation was to determine the association of maximal exercise hemodynamic responses with the risk of mortality due to all causes, cardiovascular disease (CVD), and coronary heart disease (CHD) in a population of apparently healthy individuals. The assessed population was 20,387 men and 6234 women, who were patients of a preventive medicine center in Dallas examined between 1971 and 1989. The results suggested that excessive SBP or attenuated heart rate response to maximal exercise may indicate an increased mortality risk in the studied, apparently healthy population [34].

Laukkanen, Kurl, Rauramaa, Lakka, Venäläinen, and Salonen assessed the association of SBP response to exercise with the risk of acute myocardial infarction (MI). The population was 1731 middle-aged men without a history of CHD who underwent an exercise test using a cycle ergometer. Both the rate and levels of rise in SBP during a progressive exercise test were risk predictors for acute MI [35].

Interestingly, the result of another study performed by those authors was that the population of studied men with SBP rise >19.7 mmHg per minute of exercise duration had a 2.3-fold increased risk of any stroke and a 2.3-fold increased risk of ischemic stroke compared with men whose SBP rise was <16.1 mmHg per minute [36].

Researchers from Japan concluded in their study that regardless of the presence of resting hypertension in patients with a hypertensive response to exercise, impaired longitudinal LV diastolic function and exercise intolerance exist [37].

The conclusion of a work that undertook a cross-sectional study of 3949 adolescents was that exaggerated exercise SBP is associated with higher left ventricular mass (LVM) [38].

In another study, Schultz, Hare, Marwick, Stowasser, and Sharman sought to determine whether masked hypertension (MH) could be identified while taking into consideration BP measured during a single bout of low-intensity exercise. BP was measured at rest and during short, low-intensity cycling exercise (60–70% of age-predicted maximum heart rate) in 75 untreated individuals with HRE (54 ± 9 years). All patients underwent 24 h ambulatory BP monitoring (ABPM), and MH was defined as clinical BP < 140/90 mmHg and ABPM BP ≥ 130/80 mmHg. There were 42 (56%) patients with MH, and at rest, systolic (SBP) was higher in subjects with MH compared with those without MH. During exercise, MH patients had significantly higher SBP and a greater change from baseline. Low-level exercise SBP was independently associated with MH, and if ≥175 mmHg, it identified MH with 74% sensitivity and 67% specificity. The researchers concluded that MH can be identified in untreated individuals from low-intensity exercise SBP [15].

Tsioufis et al. have shown that HRE is related to augmented albumin-to-creatinine ratio and arterial stiffness, reflecting accelerated subclinical atherosclerosis [39].

Tzemos, Lim, Mackenzie, and MacDonald, in their study assessing 82 healthy male volunteers without cardiovascular risk factors, demonstrated that exaggerated BP response to exercise is related to endothelial dysfunction, decreased proximal aortic compliance, and increased exercise-related neurohormonal activation [40].

In another study, authors from the Republic of Korea noticed impairment in longitudinal myocardial function among normotensive individuals with HRE. They claimed that HRE could cause repeated increases in afterload, resulting in subclinical myocardial dysfunction among patients without the diagnosis of hypertension [41].

In the research of Mottram, Haluska, Yuda, Leano, and Marwick, HRE, even in the absence of resting HT, was associated with subtle systolic dysfunction [42].

Keller, Stelzer, Ostad, and Post concluded that a growing number of studies support the hypothesis that HRE in cardiopulmonary exercise testing (CPET) may be a diagnostic tool allowing the identification of people at increased risk of developing arterial hypertension and future cardiovascular events [43].

Despite having such extensive knowledge about the HRE phenomenon, further research is in progress.

The above-described studies on HRE are summarized in Table 4.

## 9. Treatment and Clinical Management

Consecutive studies prove that HRE is not a benign phenomenon, but there is still no consensus on whether it should be treated in normotensive individuals [19]. What seems to be indisputable is that physical activity can positively impact endothelial function, arterial stiffness, and neurohormonal response and, as a result, contribute to improving the control of hypertension [44,45,46]. It is also a recommended preventive intervention to reduce the risk of the development of hypertension, heart failure, stroke, and other cardiovascular disease as well as their consequences.

Hare, Sharman, Leano, Jenkins, Wright, and Marwick investigated spironolactone as a treatment for abnormal vascular and myocardial stiffness in HRE. Short-term spironolactone reduced exercise BP, 24 h ambulatory BP, left ventricular mass index (LVMI), and E/e_m_ (LV filling pressure) in HRE patients without previous hypertension [47].

One RCT (randomized controlled trial) evaluated the antihypertensive efficacy and changes of neurohormonal markers of fimasartan (ARB) and atenolol (beta blocker) with exaggerated blood pressure response during exercise in essential hypertensive patients. Unfortunately, although the study is completed, no study results have been published so far.

Kim and Ha suggest that among different antihypertensive drugs that we use in daily clinical practice, the ones that could be recommended as a treatment for patients with HRE are drugs affecting the renin–angiotensin–aldosterone system: angiotensin receptor blocker (ARB) or angiotensin-converting enzyme inhibitor (ACEI). They emphasize also that beta-blockers could be a good choice considering increased sympathetic tone during exercise [19].

It remains unclear whether treating BP to guideline-recommended levels leads to the normalization of the rise in BP during exercise, which is mediated by the metaboreflex. Chant et al. aimed to assess the BP response to incremental exercise testing and metaboreflex activation in treated–controlled hypertension (*n* = 16), treated–uncontrolled hypertension (*n* = 16), and untreated hypertension (*n* = 11) and 16 control participants with normal BP (*n* = 16). The rise in absolute SBP from baseline at peak exercise was similar in controlled, uncontrolled, and untreated hypertension but greater compared with normotensive controls. Metaboreflex sensitivity was also similar in controlled, uncontrolled, and untreated hypertension but augmented compared with normotensive controls. The authors concluded that it was the first study showing that the SBP responses to dynamic exercise (both submaximal and maximal) are augmented in patients with treated–controlled hypertension and are similar to those in patients with treated but uncontrolled and untreated hypertension. As they explained, poor BP control during exercise, partially mediated by the metaboreflex, may contribute to the heightened risk of an adverse cardiovascular event even in treated–controlled patients [48].

There is no consensus on further clinical management once the phenomenon is detected. As one evaluation concluded, measuring ABPM or home BP should be considered in HRE patients [49].

## 10. Summary and Conclusions

The fact that exercise hypotension is a pathologic sign is universally accepted among health professionals [50]. More and more data deliver the information that hypertensive response to exercise is also connected to higher overall cardiovascular risk [51].

Routine blood pressure measurements during exercise tests, performed in large numbers every day in medical centers worldwide, provide far-reaching knowledge that is more extensive than previously thought. Therefore, it is important to follow the appropriate technique for measuring exercise blood pressure and to proceed further if HRE is detected.

HRE may be an early marker of cardiovascular system control disbalance that may lead to the development of hypertension and other cardiovascular diseases such as heart failure or stroke. From a clinical standpoint, it is crucial to answer the question of whether any intervention (pharmacotherapy, physical exercise training) implemented at the stage of HRE diagnosis would prevent hypertension development or reduce the risk of subsequent cardiovascular events.

## Figures and Tables

**Table 1:** Spectrum of HRE definitions [12,13].

I	SBP ≥ 210 mm Hg in men and ≥190 mm Hg in women at peak exercise intensity
II	DBP ≥ 110 mm Hg for both sexes at peak exercise intensity
III	Difference of 60 mm Hg between baseline and peak SBP for men and 50 mm Hg for women
IV	SBP ≥ 150 mm Hg in the early phase of the exercise test—at stage 1 or 2 of the Bruce protocol

SBP = Systolic Blood Pressure, DBP = Diastolic Blood Pressure.

**Table 2:** Advantages and disadvantages of both definitions of HRE (peak exercise intensity vs. moderate exercise intensity) [14,15].

	Peak Exercise Intensity-Based HRE Definition (I, II, and III in Table 1)	Moderate Exercise Intensity-Based HRE Definition (IV in the Table 1)
Advantages	The largest amount of research and conclusions regarding HRE are defined in this way. Possibly greater test sensitivity.	Possibility of assessing the BP response in a larger group of patients, including those who would not be able to achieve the more advanced stages of the Bruce protocol and, consequently, a higher level of exercise due to cardiac (chronotropic insufficiency) and non-cardiac (deconditioning, musculoskeletal disorders) limitations. Better correspondence to routine daily activities. The relatively slower speed of the treadmill during the early stages of the examination is associated with less movement of the patient’s arm and less interference with auscultation of blood pressure, allowing for a more accurate assessment.
Disadvantages	Inability of some patients to achieve advanced stages of the Bruce protocol due to cardiac and non-cardiac limitations. Worse correspondence to routine daily activities. Potential difficulties in reliably assessing blood pressure values related to movement of the patient’s arm and loudness resulting from a fast pace, e.g., a treadmill.	Limited data from previous studies.

**Table 3:** A summary of HRE data across guidelines.

Guidelines	Information on HRE
2024 European Society of Cardiology (ESC) Guidelines	YES
2017 American College of Cardiology (ACC)/American Heart Association (AHA)/AAPA/ABC/ACPM/AGS/APhA/ ASH/ASPC/NMA/PCNA Guideline for the Prevention, Detection, Evaluation, and Management of High Blood Pressure in Adults	YES
2014 Evidence-Based Guideline for the Management of High Blood Pressure in Adults—Report From the Panel Members Appointed to the Eighth Joint National Committee (JNC 8)	NO
2018 Chinese Guidelines for Prevention and Treatment of Hypertension—A report of the Revision Committee of Chinese Guidelines for Prevention and Treatment of Hypertension	NO
Japanese Society of Hypertension Guidelines for the Management of Hypertension (JSH 2019)	NO
Hypertension Canada’s 2020 Comprehensive Guidelines for the Prevention, Diagnosis, Risk Assessment, and Treatment of Hypertension in Adults and Children	NO
National Institute for Health and Care Excellence Guidelines—Hypertension in adults: diagnosis and management do not provide information on HRE	NO

**Table 4:** Summary of studies on HRE.

	Authors	Year of the Publication	Studied Population	Aim	Conclusion
1	Papademetriou, V. et al. [30]	1989	19 patients with established mild to moderate hypertension	Investigation of the relationship between resting and exercise blood pressure and catecholamines to the degree of left ventricular hypertrophy	Results suggest that systolic blood pressure achieved at low level of exercise (5 mets), corresponding to usual daily activities, may be the most important determinant of left ventricular hypertrophy in patients with hypertension
2	Ren, J. F. et al. [31]	1985	67 patients with hypertension and 19 normal subjects	Examination of the relation between left ventricular mass determined by two-dimensional echocardiography and exercise blood pressure in patients with hypertension	In patients with hypertension, left ventricular mass index is poorly related to blood pressure at rest, but is related to exercise systolic blood pressure. Patients with an exercise systolic blood pressure of 190 mm Hg or greater usually have an increased left ventricular mass
3	Schultz, M. G. et al. [14]	2016	100 patients free from coronary artery disease	HRE may be indicative of underlying hypertension unnoticed by standard clinic (resting) BP measures, but this has never been confirmed by association with hypertension defined using ambulatory BP monitoring	Irrespective of resting BP, systolic BP ≥ 150 mmHg during early stages of the Bruce exercise stress test is associated with presence of hypertension
4	Kokkinos, P. et al. [32]	2007	790 prehypertensive individuals	Determining associations between exercise blood pressure and left ventricular structure	Moderate improvements in cardiorespiratory fitness achieved by moderate-intensity physical activity can improve hemodynamics and cardiac performance in prehypertensive individuals and reduce the work of the left ventricle, ultimately resulting in lower left ventricular mass
5	Schultz, M. G. et al. [6]	2013	12 longitudinal studies with a total of 46,314 individuals without significant coronary artery disease	Providing a systematic review and meta-analysis of published literature to determine the value of exercise-related blood pressure (independent of office BP) for predicting cardiovascular events and mortality	HRE at moderate exercise intensity during exercise stress testing is an independent risk factor for cardiovascular events and mortality
6	Weiss, S. A. et al. [33]	2010	6578 asymptomatic Lipid Research Clinics Prevalence Study participants	Assessing, whether individuals with HREhave higher risk of death from cardiovascular disease	In asymptomatic individuals, HRE carried higher risk of CVD death but became nonsignificant after accounting for rest BP. Bruce stage 2 BP > 180/90 mm Hg identified nonhypertensive individuals at higher risk of CVD death
7	Kohl 3rd, H. W. et al. [34]	1996	20,387 men and 6234 women, patients of a preventive medicine center in Dallas, TX	Determining the association of maximal exercise hemodynamic responses with risk of mortality due to all causes, cardiovascular disease, and coronary heart disease	Exaggerated SBP or an attenuated heart rate response to maximal exercise may indicate an elevated risk for mortality in this apparently healthy population
8	Laukkanen, J. A. et al. [35]	2006	1731 middle-aged men without history of coronary heart disease	Assessing the association of systolic blood pressure response to exercise with the risk of an acute myocardial infarction	Both rate and levels of rise in systolic blood pressure during a progressive exercise test were risk predictors for acute myocardial infarction
9	Kurl, S. et al. [36]	2001	1026 men without clinical coronary heart disease, antihypertensive medication, or prior stroke at baseline	Studying the associations between SBP rise, percent maximum SBP at 2 min after exercise, and the risk of stroke	SBP rise during exercise and percent maximum SBP at 2 min after exercise were directly and independently associated with the risk of all stroke and ischemic stroke
10	Takamura, T. et al. [37]	2008	129 subjects with a preserved ejection fraction and a negative stress test	Assessing whether patients with a marked hypertensive response to exercise have LV diastolic dysfunction leading to exercise intolerance, even in the absence of resting hypertension	Irrespective of the presence of resting hypertension, patients with hypertensive response to exercise had impaired LV longitudinal diastolic function and exercise intolerance
11	Schultz, M. et al. [38]	2016	3949 adolescents who were part of a UK population-based birth cohort study	Determining associations of exercise BP with left ventricular mass in adolescents, with consideration of the possible confounding effect of body composition	Exaggerated exercise systolic BP is associated with higher LVM, adjustment for body composition attenuates but does not abolish this association
12	Schultz, M. G. et al. [15]	2011	75 untreated subjects with a hypertensive response to exercise	Determining whether masked hypertension could be identified from blood pressure taken during a single bout of low-intensity exercise	Masked hypertension can be identified in untreated individuals from low-intensity exercise systolic blood pressure
13	Tsioufis, C. et al. [39]	2008	171 untreated males with stage I–II essential hypertension and a negative treadmill exercise test	Investigation of the relationships between a hypertensive response to exercise and urinary albumin excretion and arterial stiffness in hypertensives	A hypertensive response to exercise is related to augmented albumin-to-creatinine ratio and arterial stiffness, reflecting accelerated subclinical atherosclerosis
14	Tzemos, N. et al. [40]	2015	82 healthy male volunteers without cardiovascular risk factors	Assessing the mechanism of how hypertensive response to exercise is generated, and how it relates to the future establishment of cardiovascular disease	A hypertensive response to exercise is related to endothelial dysfunction, decreased proximal aortic compliance, and increased exercise-related neurohormonal activation, the constellation of which may explain future cardiovascular disease
15	Yang, W. I. et al. [41]	2014	171 normotensive individuals without any structural heart disease	Comparing myocardial function between normotensive individuals with and without hypertensive response to exercise	Normotensive individuals with a hypertensive response to exercise exhibit impairment in longitudinal myocardial function
16	Mottram, P. M. et al. [42]	2004	41 patients with HRE, comprising 22 patients with hypertension and 19 without resting hypertension and 17 matched control subjects without HRE	Determining if a hypertensive response to exercise is associated with myocardial changes consistent with early hypertensive heart disease	HRE is associated with subtle systolic dysfunction, even in the absence of resting HT
17	Keller, K. et al. [43]	2017	18 original studies about EBPR in CPET, which included a total of 35,151 normotensive individuals for prediction of new onset of arterial hypertension in the future and 11 original studies with 43,012 enrolled subjects with the endpoint of cardiovascular events in the future	Assessing whether an exaggerated blood pressure response in cardiopulmonary exercise testing could help to identify seemingly cardiovascular healthy and normotensive subjects, who have an increased risk of developing arterial hypertension and cardiovascular events in the future	EBPR in CPET may be a diagnostic tool to identify subjects with an elevated risk of developing arterial hypertension and cardiovascular events in the future
